# Echinatin Ameliorates Insulin Resistance and Hepatic Lipid Accumulation in db/db Mice

**DOI:** 10.3390/biomedicines14092091

**Published:** 2026-09-17

**Authors:** Hong Xu, Lijie Jiang, Yuanjun Zhang, Jingqing Hu

**Affiliations:** 1 Department of Basic Theory of Traditional Chinese Medicine, Chengdu University of Traditional Chinese Medicine, No. 1166 Liutai Avenue, Wenjiang District, Chengdu 611137, China; 2 Institute of Basic Theory for Chinese Medicine, China Academy of Chinese Medical Sciences, No. 16 Dongzhimen Nanxiaojie, Dongcheng District, Beijing 100700, China; 3 Institute for Interdisciplinary Studies, Tianjin University of Traditional Chinese Medicine, No. 10 Poyanghu Road, Jinghai District, Tianjin 301617, China

**Keywords:** insulin resistance, fatty liver, oxidation stress, inflammation, echinatin (ECH), glucose tolerance

## Abstract

**Background:** Type 2 diabetes mellitus (T2DM) is a multifactorial metabolic disorder characterized by hyperglycemia, insulin resistance, hepatic steatosis, chronic inflammation, and oxidative damage. Echinatin (ECH), a naturally occurring chalcone compound, has shown potential metabolic regulatory activities, but its effects and underlying mechanisms in T2DM remain unclear. This study aimed to investigate the effects of ECH and its underlying mechanisms in db/db mice. **Methods:** Male db/db mice were orally administered ECH at low and high doses for 8 weeks. Glucose metabolism was evaluated by fasting blood glucose measurement and oral glucose tolerance test (OGTT). Insulin sensitivity was assessed by serum insulin levels and hepatic AKT phosphorylation. Pancreatic β-cell integrity was examined by insulin immunohistochemistry. Hepatic glucose metabolism-related genes, including *Pck1*, *G6pc*, *Gys1*, *Gys2*, *Gck*, and *Slc2a2*, were analyzed by quantitative PCR. Serum and hepatic lipid profiles, hepatic steatosis, inflammatory cytokines, and oxidative stress markers were also evaluated. **Results:** ECH treatment significantly reduced fasting blood glucose levels and improved glucose tolerance, as indicated by ECH-preserved pancreatic β-cell integrity and increased serum insulin levels. Furthermore, ECH enhanced hepatic insulin signaling, as demonstrated by an increased p-AKT/AKT ratio. At the transcriptional level, high-dose ECH significantly suppressed the expression of gluconeogenic genes *Pck1* and *G6pc*, while upregulating the glucose transporter gene *Slc2a2* without significantly altering *Gys1*, *Gys2*, or *Gck* expression. In addition, ECH reduced serum and hepatic triglyceride and total cholesterol levels, alleviated hepatic lipid accumulation by H&E and Oil Red O staining, and decreased circulating inflammatory cytokines (TNF-α and IL-1β) and oxidative markers (4-HNE and 8-OHdG). **Conclusions:** ECH treatment was associated with improvements in multiple metabolic parameters in db/db mice, including glucose homeostasis, insulin sensitivity, hepatic steatosis, and systemic inflammation and oxidative stress. These observations may provide a basis for further studies on the effects of ECH on T2DM-related glucose and lipid metabolism.

## 1. Introduction

Type 2 diabetes mellitus (T2DM) is a complex and progressive metabolic condition marked by persistent hyperglycemia resulting from impaired insulin production, insulin resistance, or a combination thereof [1,2]. Beyond abnormal glucose regulation, T2DM is increasingly recognized as a systemic metabolic disorder characterized by significant disruptions in lipid metabolism, inflammation, oxidative stress, and mitochondrial homeostasis. The International Diabetes Federation reports that around 536.6 million persons aged 20 to 79 were diagnosed with diabetes globally in 2021, with an anticipated rise to 783.2 million by 2045 [3]. China currently has the largest diabetic population globally, with the number of adults with diabetes expected to exceed 140 million by 2045 [3]. The rapid increase in T2DM prevalence is closely associated with modern lifestyle factors, including excessive energy intake, unhealthy dietary patterns, obesity, and physical inactivity, which cumulatively result in insulin resistance and metabolic disturbances [4]. The pathogenesis of T2DM involves multiple interconnected mechanisms, including impaired glucose and lipid metabolism, chronic low-grade inflammation, and oxidative stress. Sustained hyperglycemia fosters the excessive production of reactive oxygen species (ROS), which leads to oxidative damage and exacerbates the impairment of insulin signaling pathways [5,6,7]. Meanwhile, metabolic abnormalities associated with T2DM frequently result in progressive complications, such as hepatic steatosis, diabetic kidney disease, and cardiovascular disorders stemming from dyslipidemia [8,9,10]. Hepatic lipid accumulation driven by enhanced lipogenesis and impaired lipid oxidation is considered a critical feature linking insulin resistance with hepatic steatosis development [11]. Therefore, therapeutic strategies that are capable of simultaneously regulating glucose metabolism, lipid homeostasis, oxidative stress, and inflammatory responses may provide greater benefits for the prevention and treatment of T2DM-associated metabolic disorders. Current T2DM treatment focuses on lifestyle changes and medications. Dietary control, exercise, and weight management are key to metabolic health [12,13]. Numerous glucose-lowering medications, such as metformin, thiazolidinediones, α-glucosidase inhibitors, GLP-1 receptor agonists, and SGLT2 inhibitors, are commonly used in clinical practice [14]. Although these medications effectively reduce blood glucose levels and delay disease progression, they mainly target specific aspects of glucose regulation and cannot completely reverse the systemic metabolic abnormalities associated with T2DM. Moreover, long-term treatment may be accompanied by adverse effects, drug resistance, or limited efficacy in certain populations [15]. Thus, the identification of novel therapeutic agents with multi-target regulatory properties remains highly desirable. Phytochemicals from medicinal plants, owing to their broad biological activities and ability to modulate multiple pathogenic pathways, represent promising candidates for the treatment of metabolic diseases. Licorice (Glycyrrhiza species), a widely used medicinal herb, contains abundant flavonoids with reported antioxidant, anti-inflammatory, and metabolism-regulating properties [16]. Echinatin (ECH), a naturally occurring chalcone-type flavonoid, is one of the important bioactive components identified in licorice, particularly Glycyrrhiza inflata and Glycyrrhiza uralensis [16]. ECH has been shown to suppress reactive oxygen species (ROS) accumulation, inhibit lipid peroxidation, and reduce inflammatory mediators like NO, IL-6, and prostaglandin E2 (PGE2) in vitro and in vivo [17,18]. These biological effects suggest that ECH may have potential value in diseases characterized by oxidative stress and chronic inflammation. Evidence suggests that oxidative stress and inflammatory responses contribute to insulin resistance, pancreatic β-cell failure, and hepatic lipid accumulation in T2DM progression. Furthermore, licorice-derived flavonoids have been reported to regulate glucose and lipid metabolism through mechanisms involving improvement of insulin signaling, activation of AMP-activated protein kinase (AMPK)-related metabolic pathways, inhibition of inflammatory responses, and suppression of hepatic lipogenesis [9]. Therefore, ECH may potentially improve metabolic homeostasis by enhancing insulin sensitivity, reducing hepatic lipid accumulation, and alleviating oxidative stress-associated metabolic injury. However, whether ECH directly protects against T2DM-associated metabolic dysfunction and hepatic steatosis, as well as its underlying molecular mechanisms, remains largely unclear. In the present study, we employed db/db mice, a well-established genetic model of obesity-associated T2DM, to investigate the metabolic regulatory effects of ECH. We examined whether ECH could improve glucose and lipid metabolic disorders, reduce hepatic steatosis, and regulate oxidative stress and inflammatory responses. Furthermore, the potential mechanisms underlying the observed effects of ECH were systematically explored. This study provides preliminary observational evidence for the metabolic effects of ECH in this experiment.

## 2. Materials and Methods

### 2.1. Animals and Programs

Five-week-old male db/db mice were purchased from GemPharmatech Co., Ltd. (Nanjing, China). Mice were housed under controlled conditions (22–28 °C, 60% humidity, 12 h light/dark cycle) with free access to food and water. After one week of acclimatization, mice were randomly assigned into 3 groups: db/db (vehicle, 0.5% DMSO in water), db/db + ECH-L (low dose of echinatin [ECH, Solarbio, Beijing, China] 20 mg/kg/day) and db/db + ECH-H (high dose of ECH 50 mg/kg/day). The doses of ECH (20 and 50 mg/kg/day) were selected according to previously reported effective doses in mouse models, in which ECH exhibited beneficial pharmacological effects and acceptable tolerability [18,19]. All groups were fed a chow diet and received daily oral gavage. Body weight, food intake, and water intake were recorded twice weekly.

All animal tests were approved by the Animal Care and Use Committee of the Laboratory Animal Center (No. 2503-11) on 24 March 2025. Institute of Basic Theory for Chinese Medicine, China Academy of Chinese Medical Sciences. All animal procedures were performed in accordance with the institutional guidelines. Animals were handled gently and acclimatized prior to the experiments to minimize pain and distress. Oral gavage and routine handling were minimally invasive, and no surgical procedures were performed.

### 2.2. Oral Glucose Tolerance Test (Ogtt) Assay

After 8 weeks of treatment, the mice underwent an oral glucose tolerance test (OGTT). Following a 12 h fast, an oral glucose load of 1.5 g/kg was administered. Blood samples were collected from the tail vein at 0, 30, 60, 90, and 120 min after glucose administration, and blood glucose concentrations were measured using ACCU-CHEK Active test strips and a Roche glucometer (Roche Diabetes Care GmbH, Mannheim, Germany). The area under the curve (AUC) for glucose was calculated.

### 2.3. Blood Samples and Biochemical Measurements

At the end of the experiment period, mice were euthanized by cervical dislocation, blood was collected and serum was isolated. Serum triglyceride (TG) and total cholesterol (TC) levels were measured using commercially available biochemical assay kits (Nanjing Jiancheng Bioengineering Institute, Nanjing, China). Serum insulin, IL-1β, TNF-α, 4-HNE, and 8-OHdG levels were measured using commercial ELISA kits according to the manufacturer’s protocols.

### 2.4. H&E Staining

Liver tissues were fixed with 4% paraformaldehyde, embedded in paraffin, sectioned at 5 μm thickness, and stained with hematoxylin and eosin (H&E). Sections were stained with hematoxylin and eosin (H&E) and examined under a light microscope (Zeiss, Jena, Germany).

### 2.5. Oil Red O Staining

Liver tissues were fixed with 4% paraformaldehyde. We stained 10 μm-thick frozen sections with Oil Red O and counterstained with hematoxylin. Red lipid droplets were observed under a light microscope (Olympus Corporation, Tokyo, Japan) and quantified using ImageJ software (version 1.52a; NIH, Bethesda, MD, USA).

### 2.6. RT-PCR and Real-Time Quantitative PCR (qRT-PCR)

The RT reagent kit (RR047A, Takara, Dalian, China) was employed to reverse-transcribe 500 ng of total RNA into cDNA in accordance with the manufacturer’s instructions. Next, a quantitative polymerase chain reaction (qPCR) kit (AG1170, Accurate Biotechnology (Hunan) Co., Ltd., Changsha, China) was employed to analyze gene expression. Relative gene expression was calculated using the 2^−ΔΔCt^ method and normalized to the db/db group. Primer sequences are provided in Table S1.

### 2.7. Detection of Tg and Tc Levels in Liver Tissue

Liver tissue (20 mg) was homogenized in ice-cold PBS. We collected the supernatant after 10 min of centrifugation at 1000× *g*. Biochemical test kits (Nanjing, China) were used to quantify TG and TC levels per the manufacturer’s instructions.

### 2.8. Western Blotting

Tissue samples (20–30 mg) were lysed with lysis buffer (P0013, Beyotime Biotech Inc., Shanghai, China) and then centrifuged at 13,000× *g* for 15 min at 4 °C. Protein concentrations were determined using a BCA protein assay kit (23227, Waltham, MA, USA). Western blotting was performed as previously described [20]. Primary antibodies are listed in Supplementary Table S2. Protein bands were visualized using an enhanced chemiluminescence kit (Hercules, CA, USA) and quantified with ImageJ software (version 1.52a; NIH, Bethesda, MD, USA).

### 2.9. Immunohistochemistry (IHC)

Pancreatic tissues were fixed in 4% paraformaldehyde and embedded in paraffin. Immunohistochemical staining for insulin was performed as previously described [21]. Sections were incubated overnight with an anti-insulin primary antibody (Servicebio Technology Co., Ltd., GB13121, Wuhan, China, 1:800), followed by secondary antibody incubation for 1 h at room temperature. Sections were developed with DAB for 1 min and counterstained with Mayer’s hematoxylin for 10 min. Images were captured using a microscope (Zeiss, Jena, Germany); four fields per animal were photographed.

### 2.10. Statistical Analysis

Statistical analyses were performed using GraphPad Prism 9 software (San Diego, CA, USA). Data are presented as mean ± SEM. For normally distributed data, comparisons among multiple groups were performed using one-way analysis of variance (ANOVA), followed by Tukey’s post hoc test. When data did not satisfy normality assumptions, the Kruskal–Wallis test was used, followed by Dunn’s multiple comparisons test. For OGTT data, two-way repeated-measures ANOVA followed by Sidak’s multiple comparisons test was used. The F values, degrees of freedom (df), and exact *p* values for ANOVA analyses are provided in Supplementary Table S3. A *p* value < 0.05 was considered statistically significant.

## 3. Results

### 3.1. ECH Treatment Improves Glucose Tolerance in db/db Mice

**Figure 1:**
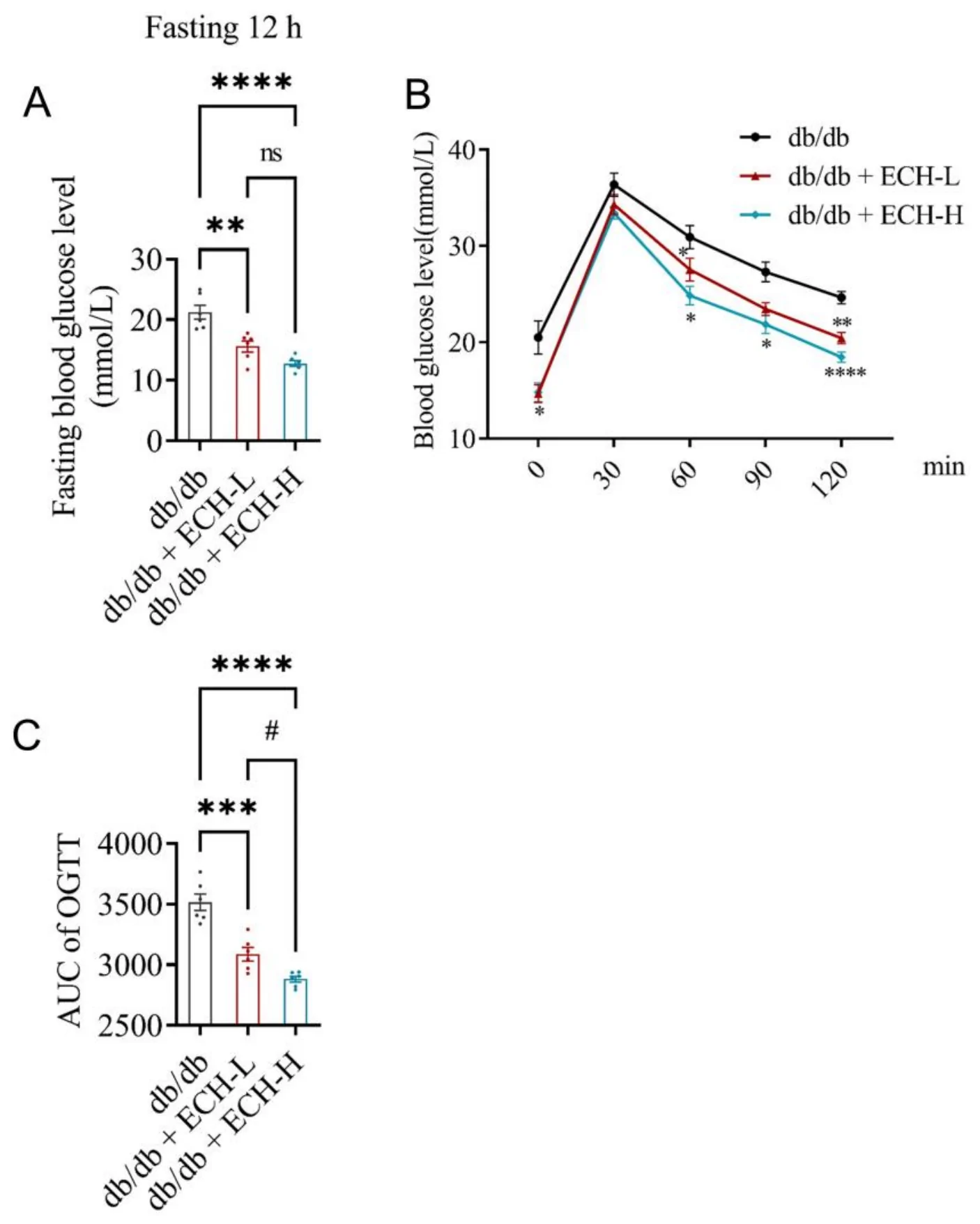
ECH improves glucose tolerance in db/db mice. (**A**) Fasting blood glucose levels after 4 weeks of treatment. (**B**) Oral glucose tolerance tests (OGTT) after 8 weeks of treatment. (**C**) Area under the curve (AUC) of the OGTT. db/db control, vehicle-treated db/db mice; ECH-L, low-dose echinatin (ECH) (20 mg/kg); ECH-H, high-dose echinatin (50 mg/kg). Data are presented as mean ± SEM (*n* = 6 mice per group). * *p* < 0.05, ** *p* < 0.01, *** *p* < 0.001, **** *p* < 0.0001 vs. db/db control; ^#^ *p* < 0.05 for ECH-H vs. ECH-L. Statistical analyses: (**A**,**C**) one-way ANOVA with Tukey’s post hoc test; (**B**) two-way repeated-measures ANOVA with Sidak’s multiple comparisons test. Two-way repeated-measures ANOVA revealed significant main effects of treatment, time, and a treatment × time interaction (all *p* < 0.0001); ns, not significant.

To evaluate the effects of ECH on glucose tolerance, fasting blood glucose (FBG) levels after 4 weeks of treatment and oral glucose tolerance tests (OGTT) were assessed after 8 weeks of treatment. Compared with the db/db control group, both ECH-L and ECH-H significantly reduced FBG levels (Figure 1A, both *p* < 0.01) and improved glucose tolerance, as demonstrated by the significant reduction in OGTT area under the curve (AUC) (Figure 1B,C, both *p* < 0.01). Notably, the ECH-H group exhibited a significantly greater efficacy compared to the ECH-L group, suggesting a beneficial effect of ECH on glucose intolerance.

### 3.2. ECH Enhances Serum Insulin Levels and Pancreatic Insulin Stores

**Figure 2:**
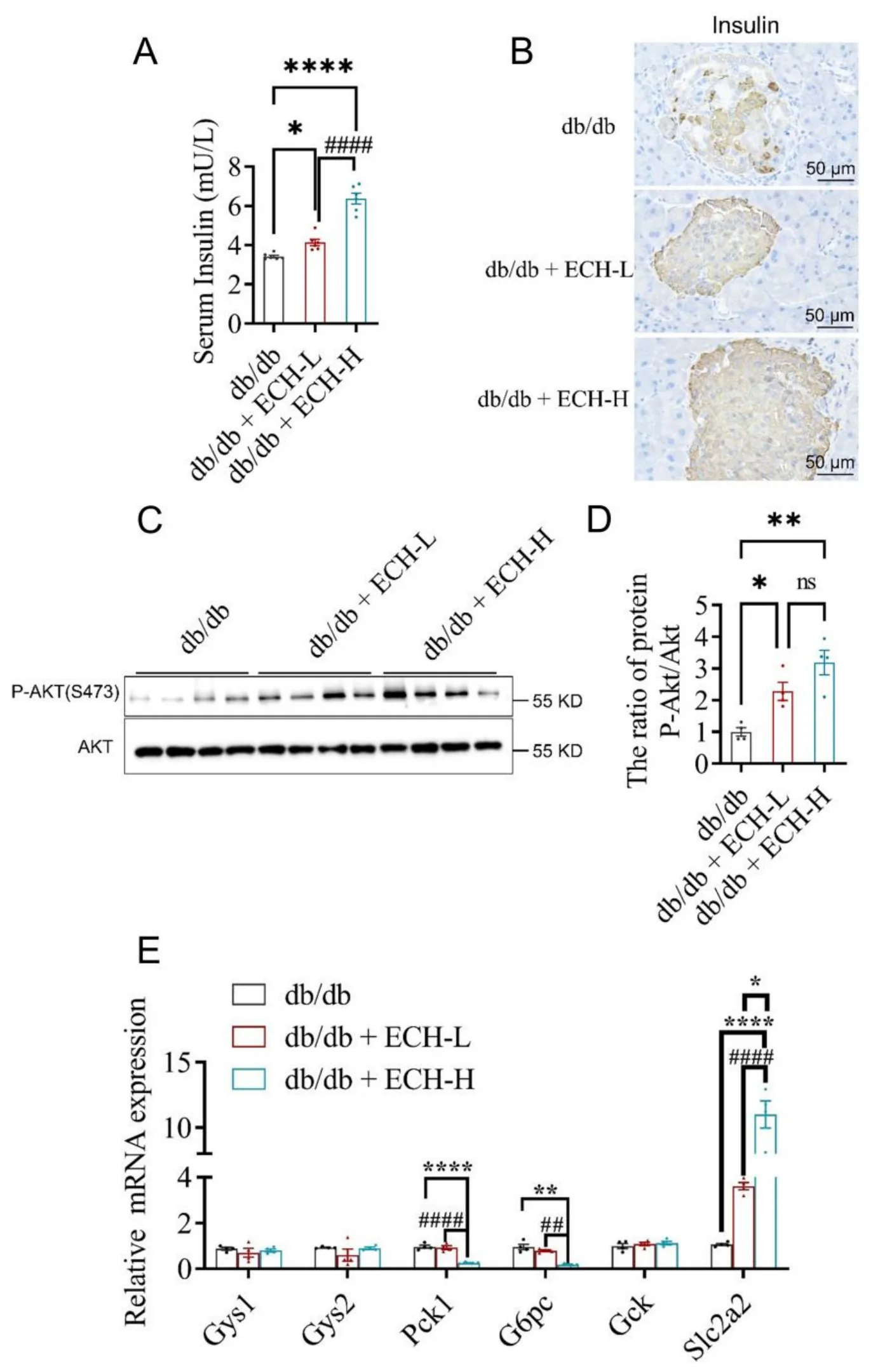
ECH improves pancreatic β-cell function and hepatic insulin signaling in db/db mice. (**A**) Serum insulin levels (*n* = 6). (**B**) Representative immunohistochemical (IHC) staining for insulin in the pancreas. (**C**) Representative Western blots of AKT and p-AKT in liver tissue. (**D**) p-AKT/AKT ratio. (**E**) Hepatic mRNA levels of *Gys1*, *Gys2*, *Pck1*, *G6pc*, *Gck*, and *Slc2a2* determined by qRT-PCR (**B**–**E**, n = 4). db/db control, vehicle-treated db/db mice; ECH-L, low-dose echinatin (ECH) (20 mg/kg); ECH-H, high-dose echinatin (50 mg/kg). Data are presented as mean ± SEM. * *p* < 0.05, ** *p* < 0.01, **** *p* < 0.0001 vs. db/db control; ^##^ *p* < 0.01, ^####^ *p* < 0.0001 for ECH-H vs. ECH-L. Statistical analyses: one-way ANOVA with Tukey’s post hoc test; ns, not significant.

Islet β-cell integrity and insulin signaling are important contributors to glucose homeostasis. To evaluate the impact of ECH on β cell function, we measured serum insulin levels and performed immunohistochemical staining for pancreatic insulin. As shown in Figure 2A,B, ECH administration significantly increased insulin levels and enhanced pancreatic insulin-positive staining compared with untreated db/db mice. Both the ECH-L (*p* = 0.0394) and ECH-H (*p* < 0.0001) groups exhibited significant increases relative to db/db controls, with the high-dose treatment yielding a substantially greater effect than the low-dose treatment (*p* < 0.0001) (Figure 2A).

To further investigate insulin sensitivity, we examined the AKT signaling pathway in the liver. ECH intervention significantly increased the hepatic p-AKT/AKT ratio in both the ECH-L (*p* = 0.0285) and ECH-H (*p* = 0.0011) groups compared with the db/db group (Figure 2C,D). However, no significant difference was observed between the ECH-L and ECH-H groups (*p* = 0.1175), suggesting that while ECH enhances hepatic insulin signaling, this effect may plateau at higher doses.

The liver plays a central role in maintaining glucose homeostasis through coordinated regulation of glucose uptake and endogenous glucose production. To delineate the molecular mechanisms underlying the glucose-lowering effects of ECH, we measured the hepatic mRNA expression of key genes involved in glycogen synthesis (*Gys1*, *Gys2*), glycolysis (*Gck*), gluconeogenesis (*Pck1*, *G6pc*), and glucose transport (*Slc2a2*, encoding GLUT2). As shown in Figure 2E, ECH treatment significantly downregulated the gluconeogenic genes *Pck1* and *G6pc*: ECH-H (*p* < 0.0001 for both) groups showed significant reductions versus db/db controls, and the suppressive effect of ECH-H was significantly greater than that of ECH-L for both genes (*p* < 0.01). In contrast, the expression of *Gys1*, *Gys2*, and *Gck* remained largely unchanged across all treatment groups (*p* > 0.05 for all pairwise comparisons), suggesting that ECH does not primarily act through modulation of glycogen synthesis or glucokinase-mediated glucose phosphorylation. Interestingly, both ECH-L and ECH-H treatment upregulated *Slc2a2* expression, with ECH-H showing a greater effect than ECH-L (ECH-L vs. db/db: *p* = 0.0372; ECH-H vs. db/db: *p* < 0.0001; ECH-H vs. ECH-L: *p* < 0.0001). Given that GLUT2, the protein encoded by *Slc2a2*, mediates bidirectional hepatic glucose transport and participates in glucose sensing, this upregulation may represent a contributing mechanism to the glucose-lowering effects of ECH.

### 3.3. ECH Ameliorates Hyperlipidemia and Hepatic Steatosis in db/db Mice

**Figure 3:**
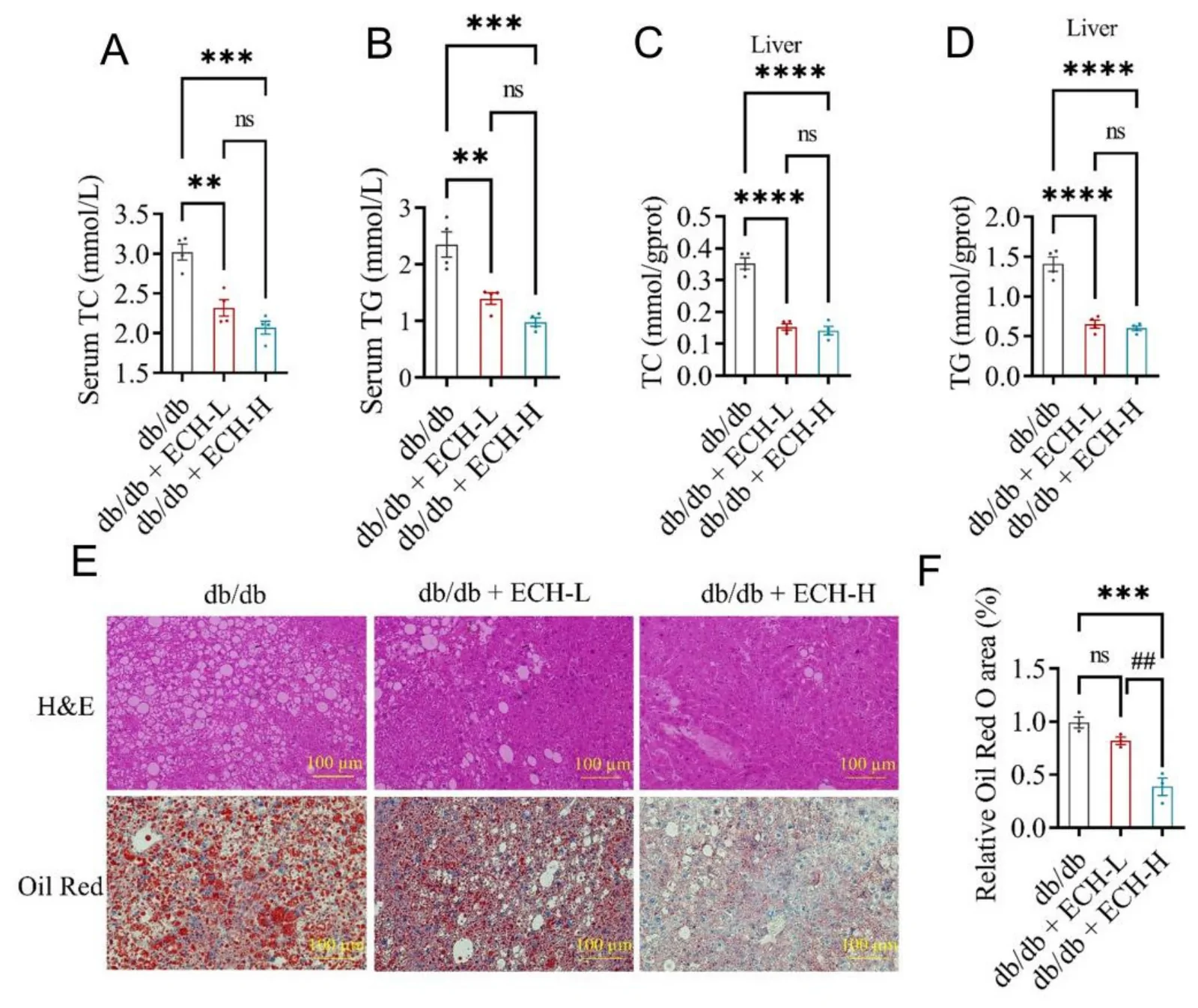
ECH attenuates hyperlipidemia and hepatic steatosis in db/db mice. (**A**) Serum total cholesterol (TC) levels. (**B**) Serum triglyceride (TG) levels. (**C**) Hepatic TC content. (**D**) Hepatic TG content. (**E**) Representative H&E and Oil Red O staining of liver sections. (**F**) Quantification of Oil Red O staining area. (**A**–**D**) *n* = 4; (**E**,**F**) *n* = 3. db/db control, vehicle-treated db/db mice; ECH-L, low-dose echinatin (ECH) (20 mg/kg); ECH-H, high-dose echinatin (50 mg/kg). Data are presented as mean ± SEM. ** *p* < 0.01, *** *p* < 0.001, **** *p* < 0.0001 vs. db/db control; ^##^ *p* < 0.01 for ECH-H vs. ECH-L. Statistical analyses: one-way ANOVA with Tukey’s post hoc test; ns, not significant.

Hyperlipidemia and nonalcoholic fatty liver (NAFLD) were observed in type 2 diabetic db/db mice [22]. To determine whether ECH intervention alleviates hyperlipidemia and hepatic steatosis, we measured serum and hepatic lipid levels and assessed hepatic lipid accumulation by histological staining after 8 weeks of treatment. As shown in Figure 3A,B, both ECH-L and ECH-H significantly reduced serum total cholesterol (TC) and triglyceride (TG) levels compared with the db/db group. ECH-L (*p* = 0.0015 for TC; *p* = 0.0035 for TG) and ECH-H (*p* = 0.0002 for TC; *p* = 0.0003 for TG) both significantly lowered serum TC and TG. However, no significant differences were observed between the ECH-L and ECH-H groups for either parameter (*p* = 0.2108 for TC; *p* = 0.1759 for TG). Similarly, hepatic TC and TG contents were significantly decreased by ECH treatment. Compared with the db/db group, both ECH-L and ECH-H markedly reduced hepatic TC (*p* < 0.0001 for both) and hepatic TG (*p* < 0.0001 for both). As observed in the serum, there was no significant difference between the ECH-L and ECH-H groups for hepatic TC (*p* = 0.8271) or hepatic TG (*p* = 0.8226, Figure 3C,D).

Histological evaluation by H&E and Oil Red O staining (Figure 3E) revealed a marked reduction in hepatic lipid accumulation following ECH treatment. Quantitative analysis of Oil Red O-positive areas (Figure 3F) showed that ECH-H significantly decreased lipid droplet deposition compared with the db/db group (*p* = 0.0009), whereas ECH-L did not produce a statistically significant reduction (*p* = 0.1818). Notably, ECH-H exhibited a significantly greater effect than ECH-L (*p* = 0.0052), demonstrating improvement in hepatic steatosis.

### 3.4. ECH Alleviates Serum Inflammation and Oxidative Stress in db/db Mice

**Figure 4:**
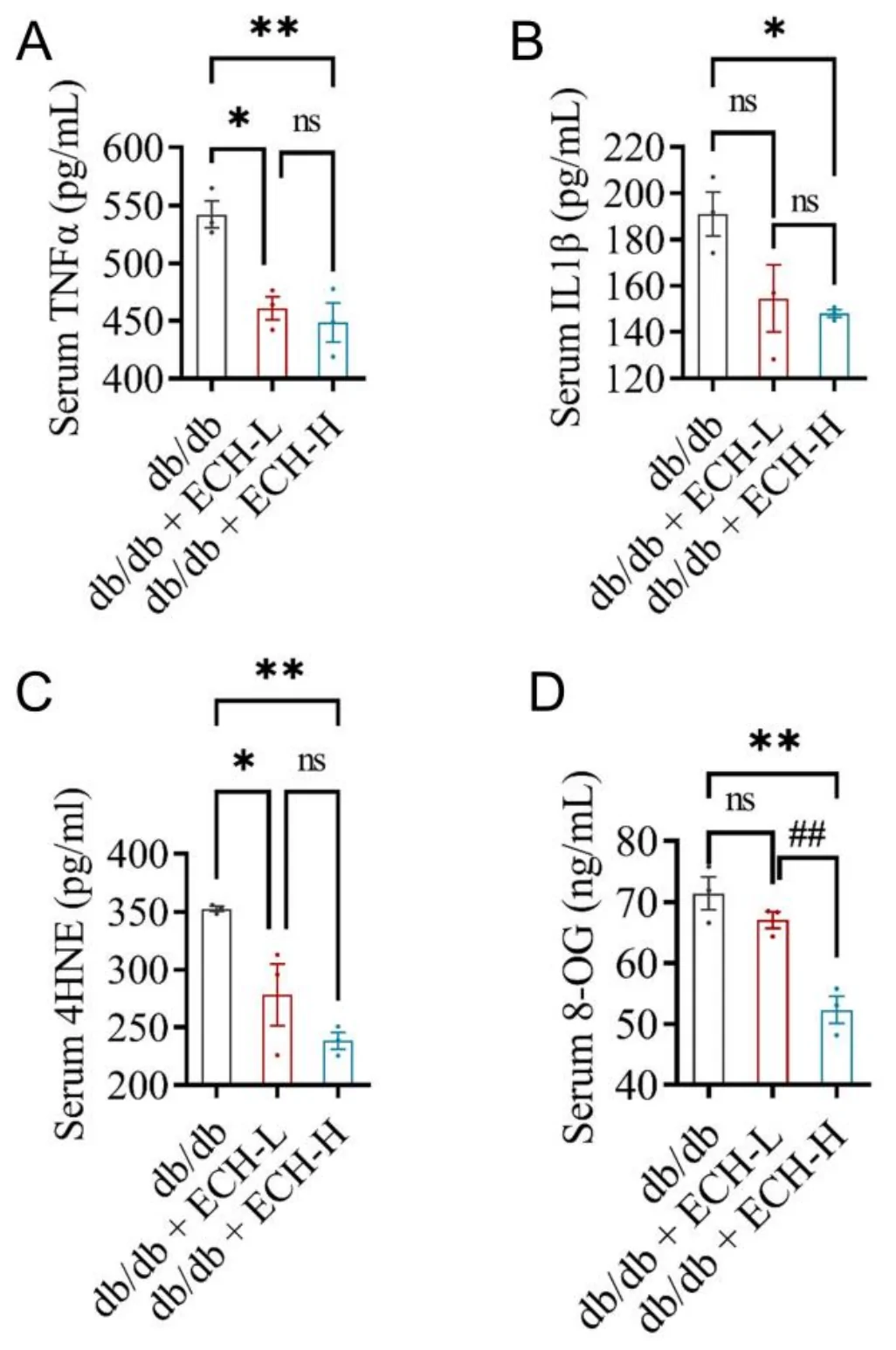
ECH alleviates serum inflammation and oxidative stress in db/db mice. (**A**) Serum tumor necrosis factor-alpha (TNF-α) and (**B**) interleukin-1 beta (IL-1β) levels. (**C**) Serum 4-hydroxynonenal (4-HNE) and (**D**) 8-hydroxy-2′-deoxyguanosine (8-OHdG) levels. db/db control, vehicle-treated db/db mice; ECH-L, low-dose echinatin (ECH) (20 mg/kg); ECH-H, high-dose echinatin (50 mg/kg). Data are presented as mean ± SEM (n = 3 mice per group). * *p* < 0.05, ** *p* < 0.01 vs. db/db control; ^##^ *p* < 0.01 for ECH-H vs. ECH-L. Statistical analyses: one-way ANOVA with Tukey’s post hoc test; ns, not significant.

To evaluate the effect of ECH on systemic inflammation, we measured serum levels of the proinflammatory cytokines tumor necrosis factor-alpha (TNF-α) and interleukin-1 beta (IL-1β). As shown in Figure 4A, both ECH-L (*p* = 0.0114) and ECH-H (*p* = 0.0058) significantly reduced serum TNF-α levels compared with the db/db group, with no significant difference between the two ECH-treated groups (*p* = 0.7923). For IL-1β (Figure 4B), ECH-H significantly reduced serum levels compared with the db/db group (*p* = 0.0409), whereas ECH-L did not produce a statistically significant reduction (*p* = 0.0735). No significant difference was observed between the ECH-L and ECH-H groups (*p* = 0.8939).

Consistent with previous reports, db/db mice exhibited elevated oxidative stress markers [23]. To investigate whether ECH alleviates oxidative damage, we measured serum levels of 4-hydroxynonenal (4-HNE), a marker of lipid peroxidation, and 8-hydroxy-2′-deoxyguanosine (8-OHdG), a marker of DNA oxidative damage. As shown in Figure 4C, both ECH-L (*p* = 0.0381) and ECH-H (*p* = 0.0056) significantly reduced serum 4-HNE levels compared with the db/db group, with no significant difference between the two doses (*p* = 0.2624). For 8-OHdG (Figure 4D), ECH-H significantly decreased serum levels (*p* = 0.0019), whereas ECH-L did not produce a statistically significant reduction (*p* = 0.3824). Notably, ECH-H exhibited a significantly greater effect than ECH-L (*p* = 0.0070), indicating attenuation of DNA oxidative damage.

## 4. Discussion

Type 2 diabetes mellitus (T2DM) is a complex metabolic disorder characterized by chronic hyperglycemia, progressive β-cell dysfunction, insulin resistance, dysregulated hepatic glucose metabolism, and metabolic inflammation [24,25]. In the present study, we demonstrated that ECH exerted significant metabolic benefits in db/db mice, including reductions in fasting blood glucose, improvement of OGTT, attenuation of dyslipidemia and hepatic steatosis, activation of hepatic AKT signaling, suppression of hepatic gluconeogenic gene expression, and alleviation of systemic inflammation and oxidative damage. These results suggest that ECH ameliorates multiple pathological processes involved in T2DM progression and may represent a promising natural compound for the management of metabolic disorders. Progressive pancreatic β-cell dysfunction is a hallmark of T2DM and is closely associated with the deterioration of glycemic control [26]. Consistent with previous reports, db/db mice in the present study exhibited severe hyperglycemia and impaired glucose tolerance [27]. ECH treatment significantly reduced fasting blood glucose levels and improved glucose tolerance. Importantly, pancreatic insulin immunostaining and serum insulin measurements suggested that ECH preserved β-cell integrity and increased insulin levels. Since the maintenance of functional β-cell mass is critical for compensatory insulin production during T2DM progression, preservation of β-cell function may represent one of the major mechanisms underlying the glucose-lowering effect of ECH. However, because insulin secretory capacity was not directly evaluated in the current study, further investigations are required to determine whether ECH directly enhances glucose-stimulated insulin secretion. Overproduction of hepatic glucose causes fasting hyperglycemia in T2DM [28]. Under physiological conditions, insulin suppresses hepatic gluconeogenesis primarily through activation of the PI3K/AKT signaling pathway [29]. Activated AKT phosphorylates and inhibits FOXO1, thereby reducing the transcription of key gluconeogenic genes, including *Pck1* and *G6pc* [29,30,31]. In the present study, ECH significantly increased hepatic AKT phosphorylation while reducing the expression of *Pck1* and *G6pc*. These findings suggest that ECH may suppress hepatic glucose production through activation of insulin-responsive signaling pathways. Notably, ECH had no significant effect on the expression of glycogen synthase genes (*Gys1* and *Gys2*), suggesting that inhibition of gluconeogenesis rather than stimulation of glycogen synthesis may represent the predominant mechanism contributing to improved glucose control. Interestingly, ECH increased hepatic *Slc2a2* expression. GLUT2 is the major glucose transporter in hepatocytes and plays a critical role in bidirectional glucose transport and hepatic glucose sensing. Therefore, the increased expression of *Slc2a2* may reflect improved hepatic glucose handling. Nevertheless, our current analysis was based on *Slc2a2* mRNA expression and did not directly determine GLUT2 protein abundance; the functional consequences of increased GLUT2 expression remain unclear and warrant further investigation. Furthermore, although changes in *Pck1* and *G6pc* expression strongly suggest reduced gluconeogenic activity, direct measurements of hepatic glucose production using tracer-based metabolic approaches will be necessary to confirm this mechanism.

Dyslipidemia and nonalcoholic fatty liver disease (NAFLD) are common metabolic complications of T2DM and contribute substantially to insulin resistance and systemic metabolic dysfunction [32,33]. In agreement with previous studies, db/db mice exhibited elevated serum and hepatic triglyceride and cholesterol levels accompanied by marked hepatic lipid accumulation [34]. ECH treatment significantly reduced both circulating and hepatic lipid contents and alleviated hepatic steatosis, as demonstrated by histological analyses. Excessive hepatic lipid accumulation promotes lipotoxicity, mitochondrial dysfunction, oxidative stress, and inflammatory signaling, thereby aggravating metabolic abnormalities [35,36]. Therefore, the improvement of hepatic steatosis observed following ECH administration may contribute not only to improved lipid homeostasis but also to enhanced glycemic control and overall metabolic health. These findings indicate that ECH exerts beneficial effects on both glucose and lipid metabolism. Accumulating evidence indicates that chronic low-grade inflammation plays a central role in the pathogenesis of T2DM [37,38]. Proinflammatory cytokines such as TNF-α and IL-1β impair insulin signaling and promote metabolic dysfunction through sustained activation of inflammatory pathways [39,40,41]. In the present study, ECH significantly reduced circulating TNF-α and IL-1β levels, suggesting attenuation of systemic inflammation. Previous studies have demonstrated that suppression of inflammatory signaling can improve glucose metabolism and delay the progression of diabetic complications [42], further supporting the potential importance of inflammation as a therapeutic target in T2DM.

Oxidative stress is another key pathogenic factor in T2DM. Excessive production of ROS induces oxidative damage to lipids, proteins, and DNA, contributing to β-cell dysfunction, insulin resistance, and tissue injury [39]. In the current study, ECH significantly reduced serum levels of 4-HNE and 8-OHdG, which are widely recognized biomarkers of lipid peroxidation and oxidative DNA damage [43], respectively. These findings indicate that ECH effectively alleviates oxidative stress in diabetic mice. Given the close interplay between oxidative stress and inflammation, the concurrent reduction in oxidative and inflammatory markers suggests that attenuation of these pathogenic processes may represent an important mechanism underlying the metabolic benefits of ECH. However, the molecular targets responsible for the antioxidant effects of ECH remain unclear and require further investigation. Recent evidence suggests that AKT signaling may also participate in redox regulation and metabolic homeostasis [44], raising the possibility that the beneficial effects of ECH involve coordinated regulation of insulin signaling, oxidative stress, and inflammation.

Several limitations of this study should be acknowledged. First, a non-diabetic control group was not included. Although db/db mice are a widely used model of T2DM and the present study was designed to evaluate the therapeutic effects of ECH under diabetic conditions, the lack of healthy control animals limits our ability to determine whether ECH treatment completely restores metabolic abnormalities to physiological levels or only partially improves diabetes-associated changes. Second, hepatic glucose production and insulin sensitivity were inferred from molecular and biochemical markers rather than directly assessed using hyperinsulinemic–euglycemic clamp studies, insulin tolerance tests, or tracer-based metabolic analyses. Third, the upstream molecular mechanisms responsible for AKT activation by ECH remain unknown. Finally, potential contributions of gut-derived mechanisms, including incretin secretion, intestinal glucose absorption, and gut microbiota modulation, were not investigated and warrant further study.

## 5. Conclusions

In summary, after 8 weeks of ECH administration, improvements were observed in hyperglycemia, insulin resistance, hepatic steatosis, systemic inflammation, and oxidative stress in db/db mice. ECH-treated groups showed preserved pancreatic β-cell function, increased serum insulin levels, and elevated hepatic p-AKT/AKT ratios, along with downregulated gluconeogenic genes (*Pck1* and *G6pc*) and upregulated Slc2a2 (*GLUT2*) expression. In addition, reductions were observed in serum and hepatic lipid levels, pro-inflammatory cytokines (TNF-α, IL-1β), and oxidative damage markers (4-HNE, 8-OHdG). These observations may provide a basis for further studies on the effects of ECH on T2DM-related glucose and lipid metabolism.

## Data Availability

The original contributions presented in the study are included in the article/Supplementary Material. Further inquiries can be directed to the corresponding author(s).

## Supplementary Materials

The following supporting information can be downloaded at https://www.mdpi.com/article/10.3390/biomedicines14092091/s1. Table S1: The sequences of primers for qRT-PCR; Table S2: The primary antibodies used in western blot analysis antibodies dilution and source; Table S3: Statistical analysis methods and data.xlsx.
