# Supplementary material for: Echinatin Ameliorates Insulin Resistance and Hepatic Lipid Accumulation in db/db Mice
Source: Biomedicines. 2026 Sep 17;14(9):2091. doi: 10.3390/biomedicines14092091 (PMC13604247; doi:10.3390/biomedicines14092091)
Supplement: Supplementary file 1 [file biomedicines-14-02091-s001.zip › Supplementary Table S1 The sequences of primers for qRT-PCR.pdf]

Supplementary Table S1. The sequences of primers for qRT-PCR.

Name	Sequences
Mouse-*Gapdh*	Forward: 5'-AGGTCGGTGTGAACGGATTTG-3'
	Reverse: 5'- TGTAGACCATGTAGTTGAGGTCA-3'
Mouse-*Gys1*	Forward: 5'- CGGTTGTCGGACTTGCTAGATTGG -3'
	Reverse:5'-CATAGGTGAAGTGGTCTGGAAAGG-3'
Mouse-*Gys2*	Forward: 5'- CCTTGGGGTGTTTCCATCGTAC -3'
	Reverse:5'- CGGAGAGGTTTGTAGTCACACTGG-3'
Mouse-*Pck1*	Forward: 5'- AGCCTGCTCCAGCTTTGA-3'
	Reverse:5'- CCCTAGCCTGTTCTCTGTGC-3'
Mouse-*G6pc*	Forward: 5'- TGCTGTGTCTGGTAGGCAAC -3'
	Reverse:5'- AGAATCCTGGGTCTCCTTGC-3'
Mouse-*Gck*	Forward: 5'- TATGAAGACCGCCAATGTGA -3'
	Reverse:5'- TTTCCGCCAATGATCTTTTC-3'
Mouse- *Slc2a2*	Forward: 5'-GTCCAGAAAGCCCCAGATACC-3'
	Reverse:5'- GTGACATCCTCAGTTCCTCTTAG -3'
