# Supplementary material for: Echinatin Ameliorates Insulin Resistance and Hepatic Lipid Accumulation in db/db Mice
Source: Biomedicines. 2026 Sep 17;14(9):2091. doi: 10.3390/biomedicines14092091 (PMC13604247; doi:10.3390/biomedicines14092091)
Supplement: Supplementary file 1 [file biomedicines-14-02091-s001.zip › Supplementary Table S2 The primary antibodies used in western blot analysis .pdf]

Supplementary Table S2. The primary antibodies used in western blot analysis antibodies dilution and source.

Antibodies	Dilution	Source
Anti-AKT	1:1000	#9272; Cell Signaling Technology, Danvers, MA
Anti-Phospho-Akt (Ser473)	1:2000	#4060; Cell Signaling Technology
